# New Isinglass Adhesive - Plaster

**Published:** 1863-02

**Authors:** E. Andrews

**Affiliations:** Professor of Surgery in the Chicago Medical College


ARTICLE VII.

### NEW ISINGLASS ADHESIVE-PLASTER.

By E. ANDREWS, M.D., Professor of Surgery in the Chicago Medical College.

The adhesive-plasters in use, consist, almost exclusively, of two kinds: the emplastrum adhæsivum of the pharmacopœia and the isinglass plaster. The advantages and disadvantages of these articles are the following:—

1st. The emplastrum adhæsivum of the pharmacopœia:—This long used plaster has the merit of being strong and cheap. Its disadvantages are, that the turpentine in its composition is apt to irritate and even blister delicate integuments. If it is kept on hand too long, it becomes dry and will not adhere; and in military practice, if the weather is cold, the impossibility of warming the strips on the field, renders the article worthless. The common idea, that it resists moisture, on account of its resinous composition, is erroneous, as it loosens from the skin with the steady application of water, as entirely as any isinglass.

As an improvement on this old standard article, we have several varieties of isinglass plaster from different manufacturers. These differ somewhat among themselves, but are all essentially alike in appearance and use. They, apparently, consist of some form of gelatine applied to a thin, delicate slip. The plasters thus made, are thin, transparent, and adhere well. Their advantages are, that they are extremely elegant, on account of their delicate transparency, that they do not change by keeping, that they adhere readily and quickly by simply moistening the surface without requiring heat, and can be as readily removed by application of water again. Moreover, they do not irritate the most delicate skin. These qualities fit them admirably for all the lighter operations of surgery; but for the heavier work, such as extension and counter-extension in fractures of the femur, and strapping in fractures of the clavicle, they are entirely worthless, for the following reasons:—

1st. The material on which the glue is laid is too fragile, and has not strength enough to sustain the tension required.

2d. The articles, as now sold, are in two small pieces. The rolls are about nine inches wide, and purport to be a yard long. but are, generally, by the villany of somebody, found to be short of even that small measure. For adhesive-strap dressing of the clavicle, two strips are required, each two and a-half inches wide and one and a-half yards in length; and for adhesive-strap counter-extension, according to my method, in fractures of the femur, two strips are required, each two yards in length and three inches in width, besides the extension-straps and the belt-strap. No such strips can be cut from the articles now sold, except by piecing them together.

3d. The expense of the article is very heavy. These little miserable patches of plaster, each one less than a-quarter of a square yard in area, are retailed to physicians at the extortionate price of a dollar each. Now, even if it were possible to use them in heavy surgery, the cost would be a serious objection. A quantity sufficient to make a solid adhesive-strap extention and counter-extention on an adult fractured femur, would come to, at least, three dollars.

For these reasons, I have requested several apothecaries to produce a new article, which should have the strength and cheapness of the old plaster, with the advantage of the gelatine surface.

Mr. Dillingham, of State Street, has been kind enough to carry his experiments to a successful issue. He has produced an article of gelatine-plaster laid on linen, which is in large rolls of about the same width as the old emplastrum adhæsivum, and which he sells at the extremely low rate of twenty-five cents a yard, being by far the cheapest and best article in the market for heavy surgical work. This plaster has strength enough for any purpose required, and adheres to the surface with unequalled tenacity. It is also perfectly adapted to the closure of ordinary incised wounds, and to every other purpose that any plaster serves. It combines the following advantages, viz., cheapness, strength, adhesiveness, and freedom from irritation.

As it is not a patent article, and there is no secret about its composition, I trust that our large manufacturers will, by and by, come down from their high stilts and consent to produce for the profession some similar preparation, capable of being used for the work we have to do, and at a price which is less preposterous than what we have heretofore paid.

